# Improving the brain image resolution of generalized q-sampling MRI revealed by a three-dimensional CNN-based method

**DOI:** 10.3389/fninf.2023.956600

**Published:** 2023-02-16

**Authors:** Chun-Yuan Shin, Yi-Ping Chao, Li-Wei Kuo, Yi-Peng Eve Chang, Jun-Cheng Weng

**Affiliations:** ^1^Department of Medical Imaging and Radiological Sciences, Chang Gung University, Taoyuan, Taiwan; ^2^Department of Computer Science and Information Engineering, Chang Gung University, Taoyuan, Taiwan; ^3^Department of Biomedical Engineering, Chang Gung University, Taoyuan, Taiwan; ^4^Institute of Biomedical Engineering and Nanomedicine, National Health Research Institutes, Miaoli, Taiwan; ^5^Institute of Medical Device and Imaging, National Taiwan University College of Medicine, Taipei City, Taiwan; ^6^Department of Counseling and Clinical Psychology, Columbia University, New York City, NY, United States; ^7^Medical Imaging Research Center, Institute for Radiological Research, Chang Gung University and Chang Gung Memorial Hospital at Linkou, Taoyuan, Taiwan; ^8^Department of Artificial Intelligence, Chang Gung University, Taoyuan, Taiwan; ^9^Department of Psychiatry, Chang Gung Memorial Hospital, Chiayi, Taiwan

**Keywords:** super-resolution convolutional neural network (SRCNN), generalized q-sampling imaging (GQI), peak signal-to-noise ratio (PSNR), structural similarity index measure (SSIM), diffusion MRI

## Abstract

**Background:**

Understanding neural connections facilitates the neuroscience and cognitive behavioral research. There are many nerve fiber intersections in the brain that need to be observed, and the size is between 30 and 50 nanometers. Improving image resolution has become an important issue for mapping the neural connections non-invasively. Generalized q-sampling imaging (GQI) was used to reveal the fiber geometry of straight and crossing. In this work, we attempted to achieve super-resolution with a deep learning method on diffusion weighted imaging (DWI).

**Materials and methods:**

A three-dimensional super-resolution convolutional neural network (3D SRCNN) was utilized to achieve super-resolution on DWI. Then, generalized fractional anisotropy (GFA), normalized quantitative anisotropy (NQA), and the isotropic value of the orientation distribution function (ISO) mapping were reconstructed using GQI with super-resolution DWI. We also reconstructed the orientation distribution function (ODF) of brain fibers using GQI.

**Results:**

With the proposed super-resolution method, the reconstructed DWI was closer to the target image than the interpolation method. The peak signal-to-noise ratio (PSNR) and structural similarity index measure (SSIM) were also significantly improved. The diffusion index mapping reconstructed by GQI also had higher performance. The ventricles and white matter regions were much clearer.

**Conclusion:**

This super-resolution method can assist in postprocessing low-resolution images. With SRCNN, high-resolution images can be effectively and accurately generated. The method can clearly reconstruct the intersection structure in the brain connectome and has the potential to accurately describe the fiber geometry on a subvoxel scale.

## 1. Introduction

### 1.1. Super-resolution image

Image quality depends on the resolution. High-resolution images can help us work with high-precision requirements, but it is difficult to obtain high-resolution images. In recent years, improving image resolution has become an important issue in the computer vision field. Among many resolution improving methods, image super-resolution is well-known. Currently, two methods are often used for image super-resolution: interpolation- and learning-based methods. The interpolation method uses the information in a low-resolution image to input values into new pixels during the super-resolution process (Keys, 1981). Common interpolation methods include neighboring, bilinear, and bicubic interpolation. By using this method, the super-resolution process is simple and fast. However, there is a disadvantage to using this method: it cannot reconstruct the high-frequency contour signal in the image. It is easily distorted in the high-resolution image. These problems can be solved by using learning methods such as the example-based method. This method obtains the mapping between the high-resolution image and its label. Pioneers used machine learning algorithms to obtain the mapping. Glasner et al. (2009) proposed a super-resolution framework through small block analysis in interpolated results and high-resolution images. Chang et al. (2004) proposed a method based on K nearest neighbor embedding. Karl et al. (2006) predicted pixel information through a support vector machine (SVM). Schulter et al. (2015) implemented a super-resolution architecture with random forest. They developed a judgment decision tree suitable for the image super-resolution regression task. Although the example-based method requires more time to obtain a higher peak signal-to-noise ratio (PSNR) image, we can successfully complete some research with precise needs.

### 1.2. Super-resolution in medical images

When the medical image is generated by the instrument, the image often contains noise. There are also some artifacts and other noise in MRIs. Poor-quality DWI affects the quality of other mappings. It is impossible to reconstruct more accurate neural connections. Trinh et al. (2014) noted that noise or blur in the image often destroys the image contrast and affects diagnostic accuracy. The super-resolution model is a commonly used medical image reconstruction method. Using similar (same organization and type) and high-quality medical images as training resources can help us build accurate models between high-resolution and low-resolution images. To accurately perform super-resolution, Dinh-Hoan Trinh et al. designed an architecture with the first layer of the architecture for training a noise removal model (Trinh et al., 2012b) and the second layer for training a super-resolution model (Trinh et al., 2012a). These research contributions to image processing help to overcome the shortcomings of imaging hardware.

### 1.3. Super-resolution diffusion MRI

During the past 20 years, many pioneers have attempted to track the brain connectome of animals (Esther, 2020). Understanding neural connections helps scientists conduct cognitive behavioral research. There are many nerve fiber intersections in the brain that need to be observed, and the size is between 30 and 50 nanometers. In the past, kissing and curvature situations in the brain connectome could not be reconstructed due to insufficient image resolution. Although diffusion spectrum imaging (DSI), q-ball imaging (QBI), and generalized q-sampling imaging (GQI) models can solve these problems, these methods are still limited by their image resolution. Improving image resolution has become an important task. With the progress of deep learning, obtaining high-resolution images with a better signal-to-noise ratio has become easier. The dilemma encountered in reconstructing the brain connectome has been overcome, and scholars have been able to complete their research with high precision requirements.

### 1.4. Convolutional neural network

Convolutional neural networks (CNNs) are widely used in computer vision fields (LeCun et al., 1989) and have become popular in image classification. In 2012, the deep learning network AlexNet won the ImageNet competition championship with a top-5 error rate of 15.4% (Cui et al., 2017) by overtaking SVM machine learning technology. Deep learning gradually replaced SVM in the ILSVRC competition. AlexNet is composed of convolution layers, which can extract features from image data. There are some pooling layers and rectified linear unit (ReLU) layers between the convolution layers. During the convolution operation, image features are obtained. Downsampling and emphasizing features are important processes. The pooling layer can complete this operation. A reduction in the number of parameters can reduce hardware usage and computing time (Krizhevsky et al., 2012). The operation of the convolutional layer involves linear transformation. When the excitation function is not used, the neural network executes only linear transformation, and the approximate ability of the network is limited. The use of ReLU can improve the nonlinear characteristics of the convolutional network (Nair and Hinton, 2010). The nonlinear function can represent the nonlinear and complex arbitrary function mapping between the input and output. With the above characteristics, CNNs can efficiently process image data.

### 1.5. Super-resolution convolutional neural network

Chao et al. proposed a super-resolution convolutional neural network (SRCNN), a neural network composed of three convolutional layers, to implement 2D image super-resolution (Dong et al., 2016). They obtained mapping between high-resolution images and low-resolution images through this structure. Pham et al. (2017) extended the 2D SRCNN to a 3D architecture. They achieved super-resolution on MRI T1 images. Although the number of training parameters increased several times due to the expansion from the 2D network to the 3D network, the resolution of the in-plane and through-plane can be increased with a 3D model. The process of 3D image super-resolution can be simplified. Compared with the implantation method, higher PSNR and higher structural similarity index measure (SSIM) images can be obtained. Lin et al. (2021) adopted a deep neural network structure in super-resolution model training for diffusion MRI. Their method is suitable for images with wide-range intensity. Chatterjee et al. (2021) adopted UNet for training a super-resolution model and reconstructed the brain connectome with DWI results.

In this work, we attempted to achieve super-resolution on diffusion MRI images. We added a residual structure to the 3D SRCNN. The extracted features and the interpolation results were averaged. With image super-resolution technology, we were eager to obtain images with the best performance index. The 3D SRCNN algorithm has the potential to reduce the bias of resolving the fiber geometry on a subvoxel scale.

## 2. Materials and methods

### 2.1. Participants and diffusion MRI data acquisition

A total of 347 participants were recruited from the Chiayi Chang Gung Hospital. Our study was approved by the Institutional Review Board of the Chang Gung Memorial Hospital, Chiayi, Taiwan. All participants were scanned by a 3 T MRI imaging system (Verio, SIEMENS, Germany) at Chiayi Chang Gung Memorial Hospital. For diffusion imaging, a single-shot, diffusion-weighted spin echo-planar imaging sequence was performed. The image acquisition parameters were as follows: repetition time (TR)/echo time (TE) = 8943/115 msec; number of excitations = 1; field of view (FOV) = 250 × 250 mm^2^; slice thickness = 4 mm; matrix size = 128 × 128; voxel size = 3.4 × 3.4 × 4 mm^3^; *b*-values = 0, 1,000, 1,500, and 2,000 s/mm^2^ in 193 total noncollinear directions. The total acquisition time of each participant was approximately 30 min.

We selected 319 subjects’ images as training data and 28 subjects’ images as test data. The maximum common factor value of each dimension in the original image was 1. During preprocessing, we attempted to increase the image matrix size from 44 × 44 × 12 to 132 × 132 × 36 voxels.

### 2.2. Experimental design

**FIGURE 1:**
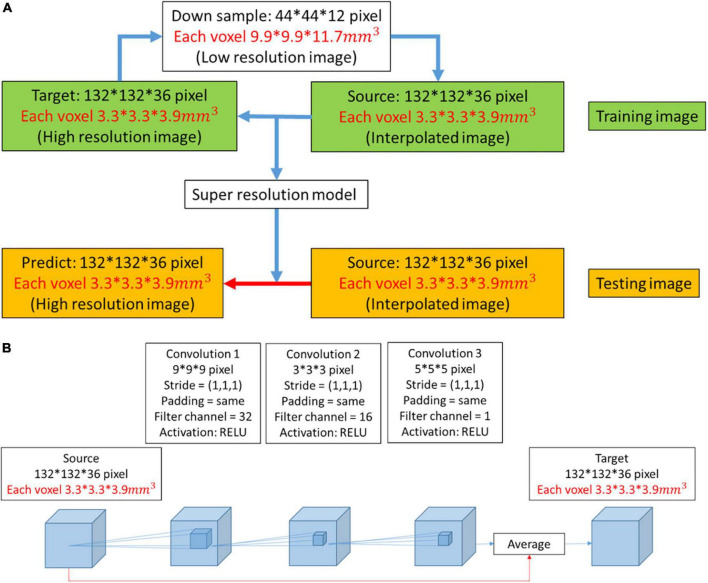
**(A)** Workflow to obtain a predicted image from a low-resolution image. When the super-resolution convolutional neural network (SRCNN) model was trained, the training pairs (source image and target image) had the same resolution. Therefore, the low-resolution image was first restored to resolution by the interpolated method (interpolated image), and the mapping between the interpolated result and the high-resolution image was obtained. The model was then applied to the testing image to obtain the predicted image (red arrow). **(B)** Three-dimensional architecture consisting of convolutional layers and pooling layers. In the red path, we used the interpolation result and feature mapping obtained by the convolutional layer to average. We used this architecture to train the super-resolution model.

In this work, we used DWI as training and testing data. The ratio of training data to test data was approximately 11:1. Initially, the high-resolution image was downsampled to a low-resolution image at a ratio of 1/3. Then, the low-resolution image was restored to resolution by the interpolated method at a ratio of 3. We attempted to obtain the mapping between the interpolated result and the high-resolution image. During the training process, the model was continuously modified with the model optimizer according to numerous pairs of interpolated results and super-resolution images. The latest model that we obtained was suitable to test images for super-resolution (Figure 1A).

### 2.3. Generalized q-sampling imaging (GQI)

Using DSI Studio, we reconstructed three GQI indices: generalized fractional anisotropy (GFA), quantitative anisotropy (QA), and normalized quantitative anisotropy (NQA) and the isotropic value of the orientation distribution function (ISO) (Yeh et al., 2010). GFA indicates the neural anisotropy measurement, QA represents the number of anisotropic spins that diffuse along fiber orientations, NQA is the normalized QA, and ISO is defined as the background isotropic diffusion (Yeh et al., 2010). We also restructured the orientation distribution function (ODF) of the low-resolution (original) image, interpolated image, super-resolution image, and high resolution (target image) using DSI Studio (Yeh et al., 2010).

### 2.4. Three-dimensional SRCNN

In this work, we trained a super-resolution model with a three-layer CNN (Figure 1B). The side lengths of the three convolutional layers were 9, 3, and 5. The filter channels of the three convolutional layers were 32, 16, and 1. The filter size and filter channel referred to the design in the 2D SRCNN (Dong et al., 2016). The larger the size of the second layer filter was, the longer the training time. However, this design improved the image reconstruction performance. There is a ReLU layer between each convolutional layer. The red line indicates that the features extracted by the convolutional layer were averaged with the interpolated image in the network.

### 2.5. Training parameter setting

In the training process, the model optimizer that we used was AdaGrad (Mukkamala et al., 2017). During the training process, the learning rate affects the reconstruction performance. AdaGrad adjusts the learning rate adaptively according to the gradient variance. If the previous gradient is small, then a faster learning rate is used for model training. The learning rate is constrained if the gradient is large in the later stage (Ward et al., 2019). This model solves the problem of reconstruction between interpolated images and high-resolution images. Therefore, we set the training batch size to 1. This allows the model to be modified according to each pairing in the training dataset. According to *b*-values, we divided all images into three groups and trained three different models. We used the corresponding model to reconstruct the test image with the same *b*-values and avoided generalizing related problems.

### 2.6. Evaluation

Performance indices, including PSNR, SSIM, and cosine similarity, were performed for evaluation. In the image reconstruction process, some parameters are used to express the degree of similarity between the reconstructed and original images. The signal-to-noise ratio (SNR) represents the ratio between the signal and noise. The PSNR is defined by the mean square error (MSE) (Tanchenko, 2014). We quantify the noise by calculating the squared deviation between the reconstructed and original images [1].


(1) $$\text{PSNR}=\frac{I_{m\,a\,x}}{\sqrt{M\,S\,E}}$$

The structural similarity index (Wang et al., 2004) is composed of three parameters: brightness*l*(*x*, *y*), contrast*c*(*x*, *y*), and structural difference*s*(*x*, *y*) (Wang et al., 2005). The degree of image similarity cannot be judged by a single view, so this indicator combines three image characteristics. The structural similarity index is the product of the three parameter values multiplied by a specific weight. The closer the value is to 1, the higher the similarity between the two images (Wang and Bovik, 2002).


(2) $$\mathrm{SSIM}\,\left(x,y\right)={\left[l\,\left(x,y\right)\right]}^{\alpha }\cdot {\left[c\,\left(x,y\right)\right]}^{\beta }\cdot {\left[s\,\left(x,y\right)\right]}^{\gamma }$$

l⁢(x,y)=2⁢μx⁢μy+C1μx2+μy2+C1

c⁢(x,y)=2⁢σx⁢σy+C2σx2+σy2+C2

$$s\,\left(x,y\right)=\frac{\sigma_{x\,y}+C_{3}}{\sigma_{x}\,\sigma_{y}+C_{3}}$$

$$C_{1}={\left(0.01\cdot \left(2^{B\,i\,t}-1\right)\right)}^{2}$$

$$C_{2}={\left(0.03\cdot \left(2^{B\,i\,t}-1\right)\right)}^{2}$$

$$C_{3}=\frac{C_{2}}{2}$$

Cosine similarity expresses the degree of similarity by the inner product between two signals. The higher the similarity of the two signals, the closer the index is to 1 (Impedovo et al., 2012).

The intensity histogram can express the distribution of pixel values in an image. The horizontal axis of the histogram represents the pixel value, and the vertical axis represents the frequency of the value band.

With the wave pattern in the histogram, we can understand the super-resolution image and the degree of similarity between the target images. We used these indicators to judge image quality. This supports naked-eye observation and allows the study to be completed from an objective perspective.

## 3. Results

### 3.1. Loss function

**FIGURE 2:**
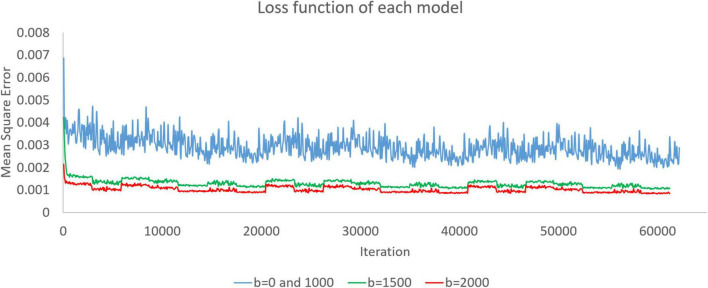
Loss functions of the *b* = 0 and 1,000, *b* = 1,500, and *b* = 2,000 models are presented as blue, green, and red curves, respectively. Since there is only one image with *b* = 0 in each subject, we used both *b* = 0 and *b* = 1,000 images to train simultaneously and obtain a model.

When we trained the super-resolution model through DWI, the model was continuously modified. In training, we recorded the loss function every time we had already trained the data of one subject. As shown in Figure 2, we recorded the loss functions of the three models during training. The model was modified according to the loss function. In this work, we set the mean square error as a loss function. The rising and falling circumstances in the loss function help us check the training dataset and optimize the training parameters.

### 3.2. Original image, interpolated image, and SRCNN image at different iterations

**FIGURE 3:**
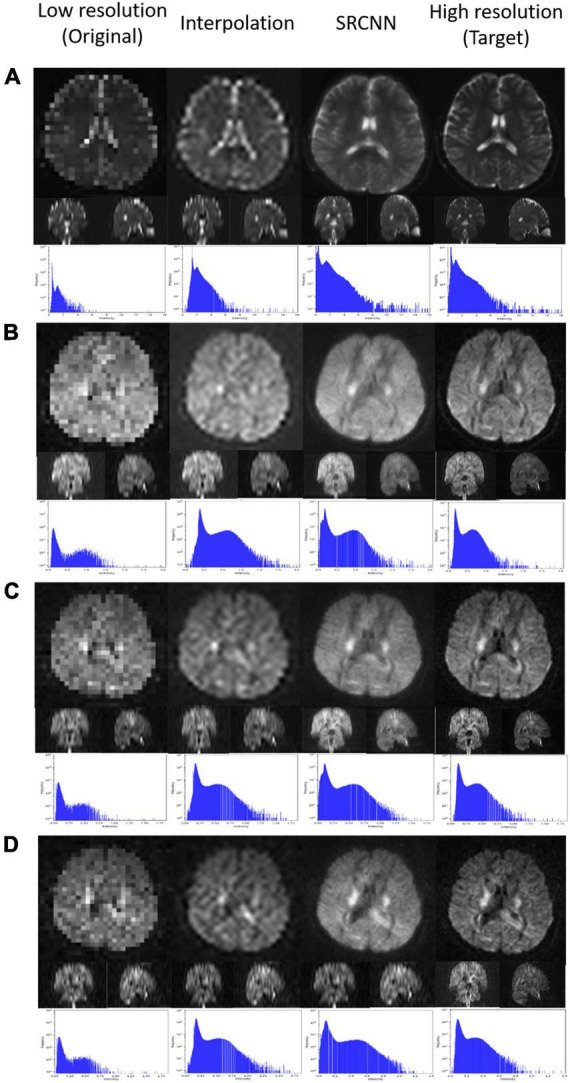
Super-resolution result of diffusion-weighted imaging (DWI) including **(A)** null; **(B)** *b* = 1,000 s/mm^2^, direction = (0.26728, –0.96286, 0.0382407); **(C)** *b* = 1,500 s/mm^2^, direction = (0.267599, –0.962729, 0.0392831); and **(D)** *b* = 2,000 s/mm^2^, direction = (0.505928, –0.834259, –0.219201).

In Figure 3, we display the DWI image super-resolution results. The histograms of the super-resolution images, low-resolution images, interpolation results, and target images shown below are compared. According to the multiplication of three indicators, we selected the best number of iterations from the three models to present reconstructed super-resolution results. Images with different *b*-values are reconstructed with their best models. There are three sections in the 3D images: axial, coronal, and sagittal views.

### 3.3. Diffusion indices

**FIGURE 4:**
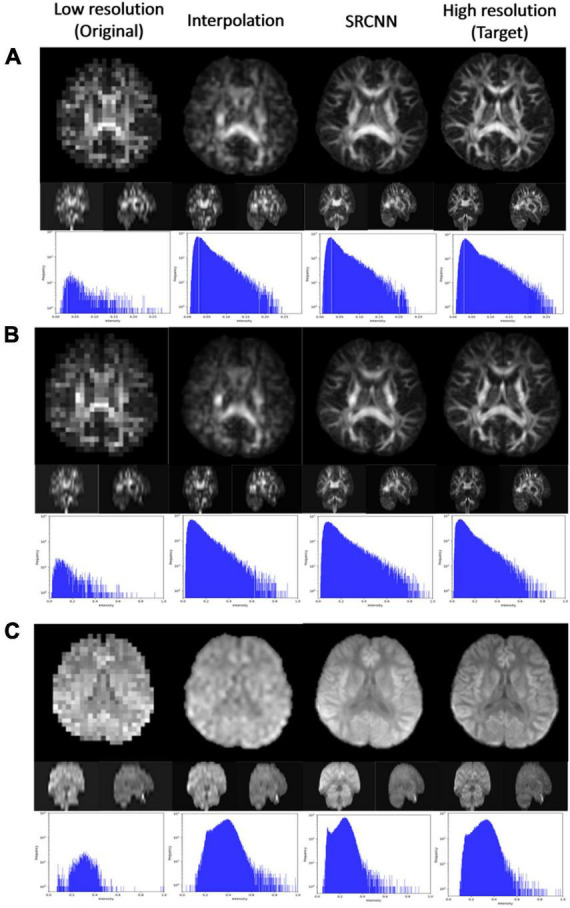
Super-resolution results of **(A)** generalized fractional anisotropy (GFA), **(B)** normalized quantitative anisotropy (NQA), and **(C)** isotropic value of the orientation distribution function (ISO).

We used DSI Studio to reconstruct three indices of GQI (GFA, NQA, and ISO) with the DWI SRCNN result, which produced the best model. In Figure 4, the results of the low-resolution image, interpolated image, super-resolution image, and target image are compared *via* three sections of axial, coronal, and sagittal views. Below each group of pictures, we used a histogram to judge the reconstruction effect.

**FIGURE 5:**
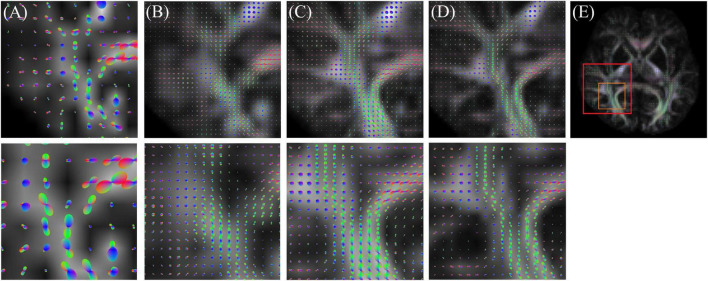
Orientation distribution function (ODF) result of **(A)** low resolution (original), **(B)** interpolation, **(C)** super-resolution convolutional neural network (SRCNN), and **(D)** high resolution (target). **(E)** Selected ROI in the whole brain.

Figure 5 shows the ODF results of the low-resolution (original) image, interpolated image, super-resolution image, and high-resolution (target image). The super-resolution image provides more detail than the low-resolution and interpolated images. Thus, ODF has the potential to assess more complex fiber connections.

### 3.4. PSNR, SSIM, and cosine similarity

**FIGURE 6:**
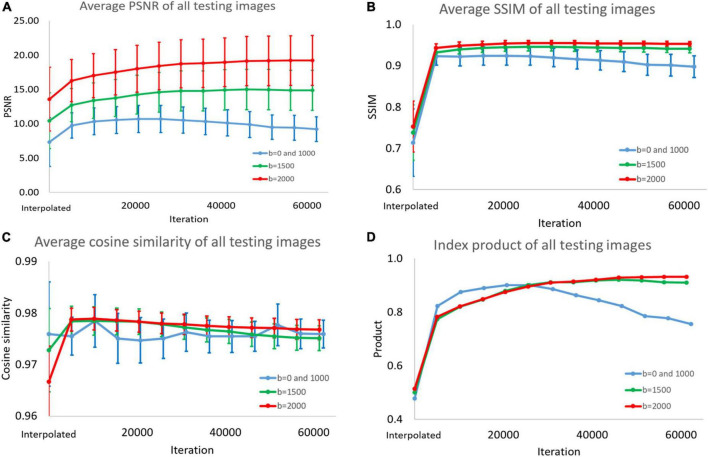
**(A)** Peak signal-to-noise ratio (PSNR), **(B)** structural similarity index measure (SSIM), **(C)** cosine similarity, and **(D)** product of the three indices. This picture shows super-resolution effect in the diffusion weighted imaging (DWI) test images. Because the peaks of the indices are different, we could not choose the best model. Therefore, we added the product of the three indicators as a standard for selection.

In Figure 6 and Supplementary Tables 1–4, we display the performance index of DWI interpolated images and super-resolution images reconstructed with the model for different numbers of iterations. We input the test images into the model for prediction every approximately 5,000 iterations. The PSNR, SSIM, and cosine similarity indices of all test results were calculated (193 < angles per subject > *28 < subjects ≥ 5,404).

The average, standard deviation, and maximum and minimum values were recorded. Finally, we multiplied the values of the three indicators. In the process, we normalized the PSNR to between 0 and 1, dividing all PSNR values by the best average value.

**FIGURE 7:**
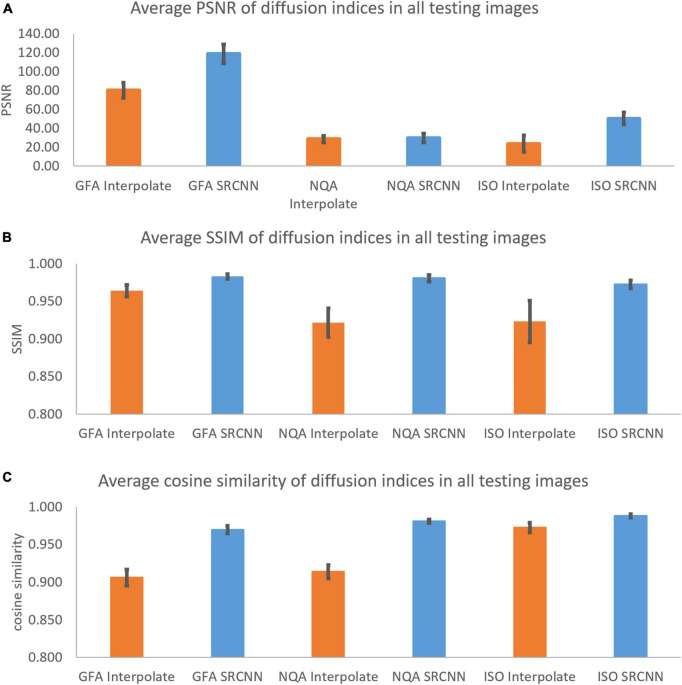
**(A)** Peak signal-to-noise ratio (PSNR), **(B)** structural similarity index measure (SSIM), and **(C)** cosine similarity of diffusion indices. These indicators are used to compare diffusion indices reconstructed with interpolation results and super-resolution results.

In Figure 7 and Table 1, we calculated the performance indicators of all images. Each mapping contains 28 images (1 < image/per subject > *28 < subjects ≥ 28). The performance indicators (PSNR, SSIM, and cosine similarity indices) of GFA, NQA, and ISO mappings were calculated and shown.

**TABLE 1:** Peak signal-to-noise ratio (PSNR), structural similarity index measure (SSIM), and cosine similarity of diffusion indices in all testing images.

PSNR	GFA interpolate	GFA SRCNN	NQA interpolate	NQA SRCNN	ISO interpolate	ISO SRCNN
Average	80.25	119.01	28.54	29.76	23.69	50.33
Std	8.06	10.00	3.71	4.64	8.87	6.55
Max	108.57	141.57	35.02	40.73	44.78	64.76
Min	62.45	104.01	20.70	21.96	10.91	33.99
**SSIM**	**GFA interpolate**	**GFA SRCNN**	**NQA interpolate**	**NQA SRCNN**	**ISO interpolate**	**ISO SRCNN**
Average	0.964	0.983	0.921	0.980	0.923	0.972
Std	0.008	0.003	0.019	0.005	0.028	0.005
Max	0.980	0.988	0.941	0.987	0.958	0.981
Min	0.940	0.972	0.853	0.966	0.834	0.959
**Cosine similarity**	**GFA interpolate**	**GFA SRCNN**	**NQA interpolate**	**NQA SRCNN**	**ISO interpolate**	**ISO SRCNN**
Average	0.906	0.970	0.914	0.981	0.972	0.988
Std	0.011	0.005	0.009	0.002	0.006	0.002
Max	0.926	0.975	0.944	0.983	0.980	0.992
Min	0.866	0.954	0.889	0.972	0.946	0.982

## 4. Discussion

### 4.1. Three-dimensional SRCNN

Converting the original 2D SRCNN model to 3D SRCNN can help improve the through-plane resolution in MRIs. If the 2D SRCNN is used to improve the through-plane resolution, then we must first train the axial, sagittal, and coronal models of MRI and then combine the test results predicted by the three models. This method is more complicated. Although there are fewer training parameters in the 2D model, the process of combining test results is more complicated. Therefore, we selected a 3D model for training, which simplifies the testing process.

### 4.2. Test image with different resolutions

**FIGURE 8:**
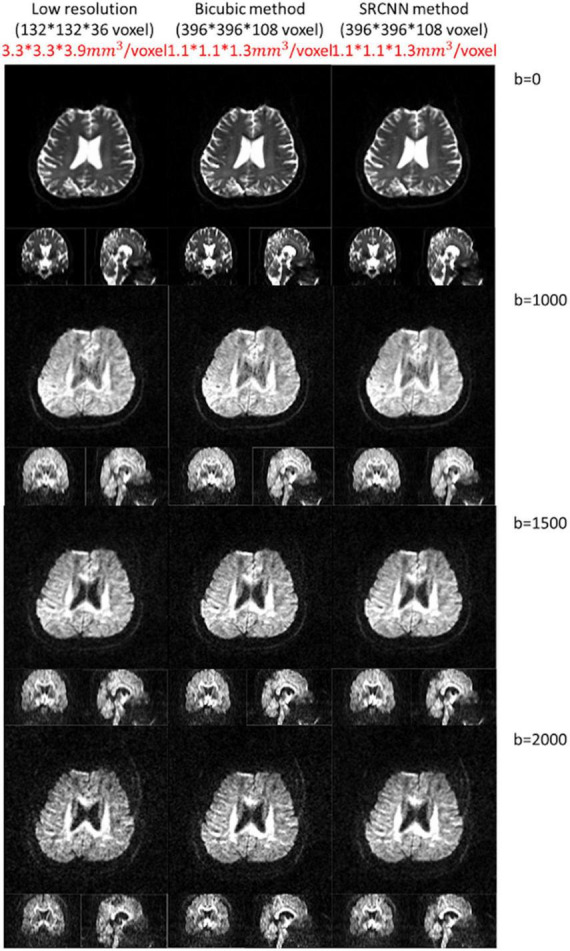
Diffusion-weighted images (DWIs) with low resolution (3.3 × 3.3 × 3.9 mm^3^) and high resolution (1.1 × 1.1 × 1.3 mm^3^). High-resolution DWIs with multiple *b*-values (0, 1,000, 1,500, and 2,000 s/mm^2^) were reconstructed by the conventional bicubic interpolation method and proposed super-resolution convolutional neural network (SRCNN) method.

This model can adapt to images with different resolutions. In the proposed deep learning structure, the training pair has the same resolution, and the model is composed of convolution filters with different values. Thus, when the model adapts images with different resolutions, convolution layers can still be used for reconstruction. We attempted to improve the DWI resolution from a voxel size of 3.3 × 3.3 × 3.9 mm^3^ to 1.1 × 1.1 × 1.3 mm^3^ with conventional bicubic interpolation and SRCNN methods. Three SRCNN models trained by the data with different *b*-values were used, and DWIs with different *b*-values were reconstructed. Compared to the bicubic method, the quality of high-resolution images with the SRCNN model significantly improved visually, as shown in Figure 8.

### 4.3. Experimental design

We initially chose a learning rate of 0.005, but a problem was found: the performance index began to decline at a lower number of iterations. This phenomenon means that the model will bounce on both sides of the best-fit point. Therefore, we reduced the initial learning rate to 0.0005, which also allowed us to judge the number of iterations where the best model is located through the curve of the performance index. The training data in our model exceeded 20,000 images, and the batch size was set to 1, resulting in more than 20,000 iterations in an epoch. Therefore, we used fewer training epochs for model training.

The obtained training data contain four types of *b*-value images of a total of 193 types of noncollinear directions. The image types contained in the training data are different. If a model is obtained with all training data, then the model must be adjusted according to each type of image. Finally, the model may cause distortion in the super-resolution result. Considering the above problems, we grouped the training data according to the *b* value and obtained the models separately.

### 4.4. Intensity normalization

Although the tissue contour super-resolution result is similar to that in the target image, the minimum value of the value range in the image will be different from that in the target image. This result affects our subsequent mapping reconstruction. Therefore, after each resolution adjustment process in the code, we performed pixel value normalization to ensure that the resulting image intensity range was the same as that of the original image.

### 4.5. Loss function

Figure 2 shows the loss functions of the three models. There are 20,735 training images in the *b* = 0 and 1,000 models. When training with batch size 1, a training cycle must go through 20,735 iterations. The loss function curves of the *b* = 0 and 1,000 models generally show a steady decline, and the curves have only small fluctuations caused by the input of different training data. There are 20,416 training images in the *b* = 1,500 and *b* = 2,000 models. When training with batch size 1, a training cycle must go through 20,416 iterations. Similarly, the loss function has some small fluctuations but continues to decline. The loss function value gradually decreases. Waves with small fluctuations are similar across all training epochs.

### 4.6. Original image, interpolated image, and SRCNN image in different iterations

Figure 3 shows the reconstruction effect of the super-resolution model on DWI. Although the interpolated method can improve the resolution of the image, this method causes some blocks to appear blurred. After using the super-resolution method proposed in this article, the reconstruction results are closer to the target. The disadvantages of interpolation can be improved.

The pattern in the histogram of the low-resolution image is similar to the pattern in the histogram of the target image. Since the number of pixels declines, the pattern looks flat. The histogram of the super-resolution result is closer to the target than the result of the interpolation method image. This was the result that we expected.

### 4.7. Diffusion indices and ODF

As shown in Figures 4, 7, the super-resolution method is better than the interpolation method. The shape of the ventricle in GFA and NQA reconstructed with SRCNN DWI looks clear.

The boundary between gray matter and white matter looks clearer. As shown in Figure 6, the ISO reconstructed with SRCNN DWI reduces the blur of the reconstruction result of the interpolation method. The ventricle and white matter area are closer to the target image. The histogram of the super-resolution image is closer to the target image than the interpolation method. The direction of the nerve fiber calculated with the super-resolution image is closer to the high-resolution (target) image.

### 4.8. PSNR, SSIM, and cosine similarity

In this study, three indices are used to quantify the super-resolution performance. These indices are then multiplied to judge the overall performance. As shown in Supplementary Table 1, the average PSNR of the *b* = 0 and 1,000 models can reach a peak at 20,735 iterations. The *B* = 1,500 and *b* = 2,000 models have the best PSNRs at the 45,932th and 61,248th iterations, respectively. After using the method proposed in this article, PSNR improved compared with the interpolation method. The *b* = 0 and 1,000 models had the best PSNR in a small number of iterations. We believe that because these training images have high contrast, it is easy to obtain clear features through the convolutional layer. This makes model training easier.

As shown in Supplementary Tables 2, 3, the three models we trained can improve the SSIM and cosine similarity of the super-resolution test image. The best SSIM and cosine similarity for the *b* = 0 and 1,000 models occurred before epoch 1. However, SSIM dropped to 0.898 in subsequent training, but the cosine similarity remained above 0.975. High-contrast images with *b* = 0 and *b* = 1,500 may also be more sensitive to model correction. Models with *b* = 1,500 and *b* = 2,000 will reconstruct the image with the best SSIM and cosine similarity at a higher number of times. The subsequent decline is slight. As shown in Supplementary Table 4, we selected the best super-resolution result based on the product. Since the PSNR change range in Supplementary Table 1 is much wider than the parameters in Supplementary Tables 2, 3, there is such a phenomenon. As shown in Figure 7, the diffusion indices reconstructed using SRCNN DWI have better performance than those reconstructed using interpolated DWI.

### 4.9. GAN comparison

In addition to SRCNN, generative adversarial networks (GANs) are another recent and state-of-the-art method for image super-resolution (Goodfellow et al., 2014). GANs consists of two neural networks: a generator network that creates high-resolution images from low-resolution ones, and a discriminator network that is trained to distinguish between real high-resolution images and synthetic ones generated by the generator. Although SRCNN, one of the pioneer methods for image super-resolution task, has shown good results, GANs has been shown to produce more realistic and high-quality images compared to SRCNN. GANs are able to generate more photo-realistic images, as well as images with finer details and textures.

However, SRCNN is a simpler architecture compared to GANs, and thus it is easier to be implemented and trained. In terms of computational cost, SRCNN requires less computational resources and time to train, making it more efficient and faster than GANs. SRCNN is a feedforward network, which means that the output of the network is a direct function of the input, making it easier to interpret and understand the network’s behavior. SRCNN has been reported to perform better on certain types of images, such as text images and medical images, where the preservation of fine details is crucial. SRCNN has less hyperparameters to tune, which makes it less sensitive to hyperparameter tuning and may lead to more robust performance in different scenarios. Overall, both SRCNN and GANs are effective methods for image super-resolution, but SRCNN is much more suitable for real-world application, especially for medical image super-resolution.

### 4.10. Limitations

Some shortcomings were not overcome in this study. Compared with the original DWI, the area around the ventricle in the SRCNN image looked brighter. This defect may affect nerve fiber reconstruction. The drawback of the expandable methodology is that the storage capacity cost and the numbers of calculations for training and prediction are proportional to the number of patches. The data form in the dataset makes the size of the dataset difficult to enlarge. If this technology is applied in clinical medicine, then the model training time needs to be reduced. We can attempt to adapt a multilayer and small area convolution layer or train the model with fewer training data.

## 5. Conclusion

In this work, we used a convolutional neural-network-based architecture to achieve DWI super-resolution. SRCNN is a more straightforward architecture that is less computationally intensive, easy to understand, performs well on medical images and is less prone to variations in hyperparameters. With this super-resolution method, the reconstructed DWI result is closer to the target image than that of the interpolation method. PSNR and SSIM were also significantly improved. The diffusion indices reconstructed by SRCNN DWI had higher performance indicators. The ventricles and white matter regions were clear. In the future, we do not need to take a long time to obtain high-resolution images. This super-resolution method can assist in postprocessing low-resolution images. We are eager to use this method to clearly reconstruct the intersection structure in the brain connectome.

## Data availability statement

The datasets presented in this article are not readily available because of the licenses/restrictions of Chang Gung Memorial Hospital, Chiayi, Taiwan. Requests to access the datasets should be directed to the corresponding author.

## Ethics statement

The studies involving human participants were reviewed and approved by the Institutional Review Board of the Chang Gung Memorial Hospital, Chiayi, Taiwan. The patients/participants provided their written informed consent to participate in this study.

## Author contributions

All authors listed have made a substantial, direct, and intellectual contribution to the work, and approved it for publication.
