# Supplementary material for: Improving the brain image resolution of generalized q-sampling MRI revealed by a three-dimensional CNN-based method
Source: Front Neuroinform. 2023 Feb 16;17:956600. doi: 10.3389/fninf.2023.956600 (PMC9978391; doi:10.3389/fninf.2023.956600)
Supplement: Supplementary file 1 [file Data_Sheet_1.docx]

**Table S1.** Average PSNR in all tests of DWI

| b=0 and 1000 model | | | | | | | | | | | | | |
| --- | --- | --- | --- | --- | --- | --- | --- | --- | --- | --- | --- | --- | --- |
| Iteration | Interpolate | 5200 | 10400 | 15600 | 20735 | 25935 | 31135 | 36335 | 41470 | 46670 | 51870 | 57070 | 62205 |
| Average | 7.33 | 9.76 | 10.35 | 10.54 | 10.68 | 10.68 | 10.53 | 10.32 | 10.11 | 9.90 | 9.50 | 9.42 | 9.21 |
| Std | 3.55 | 1.86 | 1.95 | 1.96 | 1.97 | 1.97 | 1.91 | 1.93 | 1.91 | 1.89 | 1.77 | 1.81 | 1.79 |
| Max | 19.53 | 16.52 | 17.67 | 19.56 | 20.16 | 20.40 | 19.87 | 19.97 | 19.59 | 19.24 | 18.37 | 18.77 | 18.29 |
| Min | 0.42 | 1.88 | 1.93 | 1.85 | 1.86 | 1.91 | 1.95 | 1.95 | 1.95 | 1.95 | 1.85 | 1.86 | 1.80 |
| b=1500 model | | | | | | | | | | | | | |
| Iteration | Interpolate | 5120 | 10240 | 15360 | 20416 | 25536 | 30656 | 35776 | 40832 | 45952 | 51072 | 56192 | 61248 |
| Average | 10.43 | 12.71 | 13.37 | 13.75 | 14.25 | 14.59 | 14.78 | 14.80 | 14.93 | 14.99 | 14.97 | 14.87 | 14.87 |
| Std | 4.07 | 2.42 | 2.58 | 2.69 | 2.81 | 2.88 | 2.91 | 2.92 | 2.94 | 2.94 | 2.94 | 2.93 | 2.93 |
| Max | 25.01 | 26.22 | 28.49 | 29.78 | 30.92 | 31.46 | 31.85 | 31.93 | 32.14 | 32.33 | 32.37 | 32.31 | 32.51 |
| Min | 2.85 | 8.44 | 8.81 | 8.90 | 9.06 | 9.18 | 9.25 | 9.38 | 9.60 | 9.79 | 9.90 | 9.93 | 10.04 |
| b=2000 model | | | | | | | | | | | | | |
| Iteration | Interpolate | 5120 | 10240 | 15360 | 20416 | 25536 | 30656 | 35776 | 40832 | 45952 | 51072 | 56192 | 61248 |
| Average | 13.59 | 16.27 | 17.01 | 17.50 | 18.02 | 18.43 | 18.74 | 18.81 | 18.96 | 19.13 | 19.17 | 19.21 | 19.22 |
| Std | 4.64 | 3.08 | 3.21 | 3.31 | 3.39 | 3.45 | 3.50 | 3.52 | 3.56 | 3.59 | 3.61 | 3.63 | 3.65 |
| Max | 29.60 | 31.27 | 33.30 | 34.46 | 35.34 | 35.99 | 36.49 | 36.53 | 36.66 | 36.89 | 36.99 | 37.05 | 37.18 |
| Min | 3.80 | 9.25 | 9.83 | 10.24 | 10.61 | 10.96 | 11.24 | 11.36 | 11.51 | 11.69 | 11.79 | 11.87 | 11.92 |

**Table S2.** Average SSIM in all testing DWI

| b=0 and 1000 model | | | | | | | | | | | | | |
| --- | --- | --- | --- | --- | --- | --- | --- | --- | --- | --- | --- | --- | --- |
| Iteration | Interpolate | 5200 | 10400 | 15600 | 20735 | 25935 | 31135 | 36335 | 41470 | 46670 | 51870 | 57070 | 62205 |
| Average | 0.713 | 0.923 | 0.922 | 0.924 | 0.924 | 0.923 | 0.920 | 0.916 | 0.913 | 0.910 | 0.902 | 0.902 | 0.898 |
| Std | 0.082 | 0.021 | 0.022 | 0.022 | 0.022 | 0.022 | 0.022 | 0.023 | 0.023 | 0.024 | 0.026 | 0.026 | 0.026 |
| Max | 0.859 | 0.961 | 0.961 | 0.965 | 0.965 | 0.964 | 0.963 | 0.959 | 0.959 | 0.958 | 0.953 | 0.954 | 0.952 |
| Min | 0.391 | 0.735 | 0.697 | 0.700 | 0.698 | 0.706 | 0.692 | 0.683 | 0.685 | 0.684 | 0.658 | 0.658 | 0.658 |
| b=1500 model | | | | | | | | | | | | | |
| Iteration | Interpolate | 5120 | 10240 | 15360 | 20416 | 25536 | 30656 | 35776 | 40832 | 45952 | 51072 | 56192 | 61248 |
| Average | 0.738 | 0.932 | 0.940 | 0.943 | 0.945 | 0.946 | 0.946 | 0.945 | 0.944 | 0.944 | 0.943 | 0.942 | 0.941 |
| Std | 0.067 | 0.012 | 0.011 | 0.011 | 0.010 | 0.010 | 0.010 | 0.010 | 0.010 | 0.010 | 0.010 | 0.010 | 0.010 |
| Max | 0.857 | 0.968 | 0.971 | 0.971 | 0.972 | 0.970 | 0.969 | 0.968 | 0.968 | 0.967 | 0.966 | 0.965 | 0.964 |
| Min | 0.431 | 0.872 | 0.888 | 0.895 | 0.899 | 0.901 | 0.901 | 0.900 | 0.899 | 0.898 | 0.898 | 0.896 | 0.894 |
| b=2000 model | | | | | | | | | | | | | |
| Iteration | Interpolate | 5120 | 10240 | 15360 | 20416 | 25536 | 30656 | 35776 | 40832 | 45952 | 51072 | 56192 | 61248 |
| Average | 0.752 | 0.943 | 0.949 | 0.952 | 0.954 | 0.955 | 0.955 | 0.955 | 0.955 | 0.954 | 0.954 | 0.953 | 0.953 |
| Std | 0.062 | 0.010 | 0.009 | 0.008 | 0.007 | 0.007 | 0.007 | 0.006 | 0.006 | 0.006 | 0.006 | 0.006 | 0.006 |
| Max | 0.869 | 0.969 | 0.973 | 0.973 | 0.973 | 0.972 | 0.973 | 0.973 | 0.973 | 0.973 | 0.973 | 0.972 | 0.972 |
| Min | 0.488 | 0.903 | 0.914 | 0.920 | 0.922 | 0.923 | 0.923 | 0.923 | 0.923 | 0.922 | 0.922 | 0.921 | 0.920 |

**Table S3.** Average cosine similarity in all tests of DWI

| b=0 and 1000 model | | | | | | | | | | | | | |
| --- | --- | --- | --- | --- | --- | --- | --- | --- | --- | --- | --- | --- | --- |
| Iteration | Interpolate | 5200 | 10400 | 15600 | 20735 | 25935 | 31135 | 36335 | 41470 | 46670 | 51870 | 57070 | 62205 |
| Average | 0.976 | 0.975 | 0.978 | 0.975 | 0.975 | 0.975 | 0.976 | 0.975 | 0.975 | 0.976 | 0.978 | 0.976 | 0.976 |
| Std | 0.010 | 0.004 | 0.005 | 0.005 | 0.004 | 0.004 | 0.004 | 0.003 | 0.003 | 0.003 | 0.004 | 0.003 | 0.003 |
| Max | 0.992 | 0.986 | 0.990 | 0.988 | 0.988 | 0.988 | 0.989 | 0.988 | 0.988 | 0.988 | 0.989 | 0.988 | 0.988 |
| Min | 0.885 | 0.966 | 0.966 | 0.964 | 0.964 | 0.965 | 0.965 | 0.965 | 0.965 | 0.965 | 0.968 | 0.966 | 0.966 |
| b=1500 model | | | | | | | | | | | | | |
| Iteration | Interpolate | 5120 | 10240 | 15360 | 20416 | 25536 | 30656 | 35776 | 40832 | 45952 | 51072 | 56192 | 61248 |
| Average | 0.973 | 0.978 | 0.978 | 0.978 | 0.978 | 0.978 | 0.977 | 0.977 | 0.976 | 0.976 | 0.975 | 0.975 | 0.975 |
| Std | 0.008 | 0.003 | 0.003 | 0.003 | 0.002 | 0.002 | 0.002 | 0.002 | 0.002 | 0.002 | 0.002 | 0.002 | 0.002 |
| Max | 0.989 | 0.986 | 0.986 | 0.986 | 0.986 | 0.985 | 0.985 | 0.984 | 0.984 | 0.984 | 0.984 | 0.984 | 0.983 |
| Min | 0.920 | 0.971 | 0.972 | 0.972 | 0.972 | 0.972 | 0.971 | 0.971 | 0.971 | 0.970 | 0.969 | 0.969 | 0.969 |
| b=2000 model | | | | | | | | | | | | | |
| Iteration | Interpolate | 5120 | 10240 | 15360 | 20416 | 25536 | 30656 | 35776 | 40832 | 45952 | 51072 | 56192 | 61248 |
| Average | 0.967 | 0.979 | 0.979 | 0.979 | 0.978 | 0.978 | 0.978 | 0.977 | 0.977 | 0.977 | 0.977 | 0.977 | 0.977 |
| Std | 0.007 | 0.002 | 0.002 | 0.002 | 0.002 | 0.002 | 0.002 | 0.002 | 0.002 | 0.002 | 0.002 | 0.002 | 0.002 |
| Max | 0.986 | 0.984 | 0.984 | 0.984 | 0.984 | 0.984 | 0.984 | 0.983 | 0.983 | 0.983 | 0.983 | 0.983 | 0.983 |
| Min | 0.932 | 0.972 | 0.972 | 0.972 | 0.972 | 0.972 | 0.972 | 0.972 | 0.972 | 0.971 | 0.971 | 0.971 | 0.971 |

**Table S4.** Product of three indices in DWI

| Iteration | b=0 and 1000 | Iteration | b=1500 | Iteration | b=2000 |
| --- | --- | --- | --- | --- | --- |
| 0 | 0.478 | 0 | 0.500 | 0 | 0.514 |
| 5200 | 0.822 | 5120 | 0.773 | 5120 | 0.782 |
| 10400 | 0.875 | 10240 | 0.820 | 10240 | 0.822 |
| 15600 | 0.889 | 15360 | 0.846 | 15360 | 0.848 |
| 20735 | 0.901 | 20416 | 0.879 | 20416 | 0.875 |
| 25935 | 0.900 | 25536 | 0.900 | 25536 | 0.895 |
| 31135 | 0.886 | 30656 | 0.911 | 30656 | 0.910 |
| 36335 | 0.863 | 35776 | 0.911 | 35776 | 0.913 |
| 41470 | 0.844 | 40832 | 0.918 | 40832 | 0.920 |
| 46670 | 0.823 | 45952 | 0.921 | 45952 | 0.928 |
| 51870 | 0.785 | 51072 | 0.918 | 51072 | 0.930 |
| 57070 | 0.777 | 56192 | 0.911 | 56192 | 0.931 |
| 62205 | 0.756 | 61248 | 0.910 | 61248 | 0.931 |
